# The UK’s Global Health Respiratory Network: Improving respiratory health of the world's poorest through research collaborations

**DOI:** 10.7189/jogh.09.020104

**Published:** 2019-12

**Authors:** Aziz Sheikh, Harry Campbell, Dominique Balharry, Peymané Adab, Mauricio L Barreto3, Linda Bauld, Philip Cooper, Alvaro Cruz, Fiona M Davidson, Peter Dodd, Alexandra Enocson, Neil Fitch7, Chris Griffiths8, Jonathan Grigg8, Robert S Heyderman, Rachel Jordan, S Vittal Katikireddi10, Steven Kuo, Brenda Kwambana-Adams, Alastair H Leyland10, Kevin Mortimer11, Gioia Mosler, Angela Obasi11, Mark Orme, Anne Readshaw1, Martina Savio11, Kamran Siddiqi1, Dimitra Sifaki-Pistolla7, Sally Singh, Bertie Squire11, Ioanna Tsiligianni7, Siân Williams7

**Affiliations:** 1 Usher Institute, The University of Edinburgh, Edinburgh, UK; 2 University of Birmingham, Birmingham, UK; 3 CIDACS, Salvador, Brazil; 4 St George’s University of London, London, UK; 5 Federal University of Bahia, Salvador, Brazil; 6 School of Health and Related Research, University of Sheffield, Sheffield, UK; 7 International Primary Care Respiratory Group (IPCRG); 8 Queen Mary University of London, London, UK; 9 University College London, London, UK; 10 MRC/CSO Social and Public Health Sciences Unit, University of Glasgow, Glasgow, UK; 11 Liverpool School of Tropical Medicine, Liverpool, UK; 12 University of Leicester, Leicester, UK; 13 Department of Health Sciences, The University of York, York, UK; * On behalf of the NIHR Global Health Research Unit on Respiratory Health (RESPIRE); † On behalf of the GCRF funded Tobacco Control Capacity Programme (TCCP); ‡ On behalf of the NIHR Global Health Research Group on Global COPD in Primary Care (Breathe Well); § On behalf of the NIHR Global Health Research Group to Address Smokeless Tobacco and build Research capacity in South Asia (ASTRA); ‖ On behalf of the NIHR Global Health Research Group on Asthma Attacks Causes and Prevention Study in Urban Latin America (ATTACK); ¶ On behalf of the NIHR Global Health Research Group on Social Policy and Health Inequalities; ** On behalf of the NIHR Global Health Research Unit on Mucosal Pathogens (MPRU); †† On behalf of the NIHR Global Health Research Unit on Lung Health and TB in Africa (IMPALA); ‡‡ On behalf of the NIHR Global Health Research Group on Achieving Control of Asthma in Children In Africa (ACACIA); §§ On behalf of the NIHR Global Health Research Group on Respiratory Rehabilitation (Global RECHARGE); ‖‖ Coordinators of the Global Health Research Network (GHRN)

Respiratory disorders are responsible for considerable morbidity, health care utilisation, societal costs and approximately one in five deaths worldwide [1-4]. Yet, despite this substantial health and societal burden – which particularly affects the world’s poorest populations and as such is a major contributor to global health inequalities – respiratory disorders have historically not received the policy priority they warrant. For example, despite causing an estimated 1000 deaths per day, less than half of the world’s countries collect data on asthma prevalence (http://www.globalasthmareport.org/). This is true for both communicable and non-communicable respiratory disorders, many of which are either amenable to treatment or preventable.

**Figure 1:**
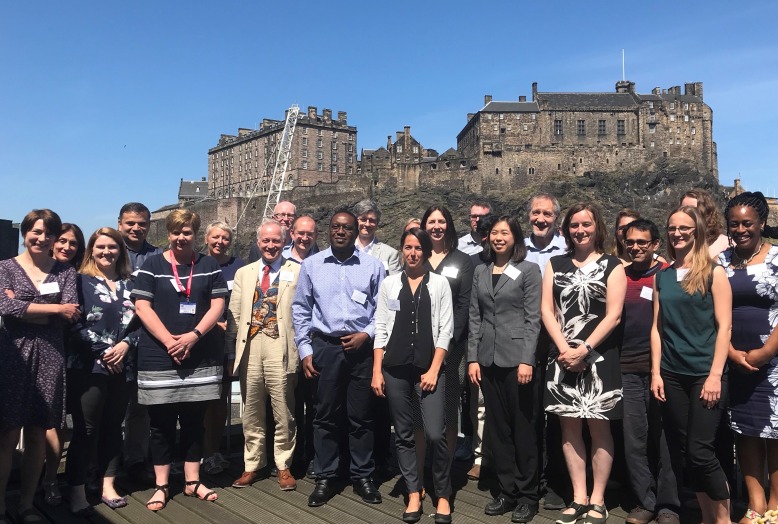
Members of the GHRN at the inaugural meeting in Edinburgh, 2018.

Part of the reason for this has been competing donor priorities leading to a focus on other conditions such as HIV, with a relative neglect of non-communicable lung diseases and of environmental determinants of lung disease. We hope that the launch of the UK’s Global Health Respiratory Network (GHRN; **Figure 1**) represents a change in approach, at least in the UK. Central to the strength of this Network is bringing together experts across the full range of respiratory health disciplines, including social, health, clinical and biomedical sciences as well as laboratory-based, clinical, implementation and health systems research, in non-communicable respiratory disorders (eg, asthma, chronic obstructive pulmonary disease [COPD]), communicable respiratory diseases (eg, tuberculosis [TB], pneumonia), and lung health (public health) to work collaboratively and synergistically, rather than competitively. It also brings those working in public health, primary care, secondary care (paediatrics and adult) and palliative care together, looking at respiratory diseases through a system-wide scope to – most importantly – lead to improvements in the respiratory health of the world’s poorest people.

Following the 2015 decision of the UK Government to channel £1.5 billion of the Official Development Assistance (ODA) budget through UK Research and Innovation (UKRI)[5], there was an open call for proposals from the UK’s National Institute for Health Research (NIHR) to establish Global Health Research Groups and Units [https://www.nihr.ac.uk/explore-nihr/funding-programmes/global-health/]. Groups would represent new partnerships between UK and low- and middle-income country (LMIC) partners, and Units would be for existing UK-LMIC partnerships to scale-up their work. Both have strong, equitable north-south partnerships that focus on addressing the needs of the poorest populations in hitherto neglected disease areas. In response to this NIHR initiative, and a related Global Challenges Research Fund (GCRF) programme from UKRI, a number of proposals with a focus on, or including, respiratory health were successful, as summarised in **Table 1**.

**Table 1:** Global health initiatives participating in the GHRN

Unit/Group/Project	Lead UK Organisation	Partner countries
NIHR Global Health Research Unit on Respiratory Health (RESPIRE); www.ed.ac.uk/usher/respire	University of Edinburgh	Bangladesh, India, Malaysia, Pakistan
NIHR Global Health Research Unit on Lung Health and TB in Africa (IMPALA); www.lstmed.ac.uk/impala	Liverpool School of Tropical Medicine	Cameroon, Ethiopia, Ghana, Kenya, Malawi, Nigeria, South Africa, Sudan, Tanzania, Uganda
NIHR Global Health Research Unit on Mucosal Pathogens (MPRU); www.mpru.org/research	University College London	Ghana, Kenya, Malawi, Mali, South Africa, The Gambia, Uganda
NIHR Global Health Research Group on Social Policy and Health Inequalities; www.gla.ac.uk/sphi	University of Glasgow	Brazil
NIHR Global Health Research Group on Global COPD in Primary Care (Breathe Well) www.birmingham.ac.uk/research/activity/applied-health/research/breathe-well	University of Birmingham	Brazil, China, Republic of North Macedonia, Georgia
NIHR Global Health Research Group on Achieving Control of Asthma in Children In Africa (ACACIA); https://www.acacia-asthma.org	Queen Mary University of London	Ghana, Malawi, Nigeria, South Africa, Uganda, Zimbabwe
NIHR Global Health Research Group on Addressing Smokeless Tobacco and building Research capacity in South Asia (ASTRA); www.york.ac.uk/igdc/research/astra-project	University of York	Bangladesh, India, Pakistan
NIHR Global Health Research Group on Respiratory Rehabilitation (Global RECHARGE); https://www.leicesterbrc.nihr.ac.uk/themes/respiratory/research/global-recharge	University of Leicester	India, Kyrgyzstan, Sri Lanka, Uganda
NIHR Global Health Research Group on Asthma Attacks Causes and Prevention Study in Urban Latin America (ATTACK); www.sgul.ac.uk	St George's, University of London	Brazil, Ecuador
UKRI GCRF: A mathematical modelling framework for tuberculosis burden estimation and economic evaluation of pharmaceutical interventions	University of Sheffield	Malawi, South Africa, Zambia
UKRI GCRF: Tobacco Control Capacity Programme (TCCP); http://ghpu.sps.ed.ac.uk/gcrf-tccp	University of Edinburgh	Bangladesh, Ethiopia, India, Ghana, Pakistan, South Africa, The Gambia, Uganda

COPD – chronic obstructive pulmonary disease, GHRN – Global Health Respiratory Network, NIHR – National Institute for Health Research

This has created a cohort of UK-funded Southern-facing research collaborations with a shared focus not only on respiratory related illness, but on capacity building and multidisciplinary research in active partnership with LMIC researchers. In recognition of the opportunity to move towards a more collaborative model of working, a number of the funded programmes addressing respiratory diseases came together with a view to sharing experiences, knowledge, lessons learnt and networks; leading to the formation of the GHRN. The core aim of the GHRN is to work collaboratively to improve global respiratory health, focusing on the poorest populations of the world. To this end, GHRN members, with funders’ approval, agreed to pool resources to create a secretariat.

The UK arm of the GHRN has met both virtually and in-person to share ideas and establish initial priorities for work over the short- to medium-term. This initial list of priorities is summarised in **Table 2**. More fundamentally, we have sought to develop respectful, trusting relationships between the scientific and management leadership teams of these various initiatives that enable collaborative sharing and learning. Examples have included supporting ethics and regulatory approvals for overseas studies where a suitable infrastructure does not exist in an institution, sharing experiences in relation to good financial grant practice, and identifying additional networks through which pathways to impact can be accelerated and amplified.

**Table 2:** Agreed initial priorities for the GHRN

Deliverable	Initial prioritisation
Map and describe briefly each partner’s relevant research - provide the baseline.	High
Include categories such as: Countries, respiratory category (eg, tobacco dependence, asthma, COPD, pneumonia, TB); study design (eg, RCT, cross-sectional study), what inequalities are being considered (eg, gender, income, employment), cross-cutting themes.	
Heat map showing intensity of GHRN research activity overlaid on burden of respiratory diseases (prevalence and DALYs of lower respiratory infections, asthma, COPD, TB, lung cancer).	High
Heat map showing intensity of research activity compared to burden of: a) respiratory diseases; and b) key behavioural and environmental exposures, including tobacco dependence, indoor air pollution and outdoor air pollution.	High
Summary of capacity building initiatives, including but not limited to, training resources available eg, spirometry, patient and public involvement, qualitative research, stakeholder mapping.	High
Register of data sets created by the Groups/Units that could be used in further studies.	High
Programme of high-level engagement opportunities.	High
Develop a national networking event to agree research ideas to address knowledge gaps identified by mapping and by policymakers.	High
Map health inequalities: ideally the burden faced by the poorest in society in each country which, seen alongside the GBD maps, would show where the effort needs to be concentrated.	Medium
Initially - scope what data exist and in which countries.	
Analysis of policy and engagement activities with policy influencers eg, parliamentarians, civil servants, non-governmental organisations, international agencies; other effective advocacy organisations and individuals – with the aim to identify areas of harmonisation, gaps, key messages, to facilitate joint planning and working.	Medium
Summary of tools used to engage policymakers and policy influencers eg, mapping tools, tools to analyse enablers and barriers, successful engagement activities and their costs, success stories, policy briefs, fact sheets, infographics, links to promotional videos.	Medium
Coordinate submissions for one or two collaborative commentaries.	Medium
Proposals for joint symposia/workshops at upcoming conferences.	Medium
Intelligence gathering to make the most of existing opportunities for networking for PhD students across the Consortium.	Review – 3 monthly

DALYs – disability-adjusted life-years, RCT – randomised controlled trial, TB - tuberculosis

Whilst it is still early days for the GHRN, we are pleased to see that our collaborative approach has been welcomed by our funders and, as such, other Units and Groups are being recommended to consider this approach. We hope that this will lead to researchers in other disease areas following suit to seek out synergies that can accelerate collaborative global working in their respective fields.

Although encouraged by the opportunities for working together, there are of course challenges. For example, within the limited resources available, we are grappling with how best to ensure that researchers based in LMICs are able to lead and shape this collaboration. Building on the equitable partnerships developed and nurtured as part of these initiatives, we are looking into the practicalities of using web-based platforms to facilitate real-time engagement across our Network; this is not straightforward as we have yet to identify a platform that works across all countries and there are also logistical and financial challenges of organising simultaneous interpreting facilities across multiple languages. However, we are confident that by working together we can greatly increase our ability to overcome these challenges.

Looking ahead, we see tremendous opportunities from joined-up working. Ours is most definitely an open model of collaboration that will, we hope, see the GHRN grow in the coming years. We aspire, with continuing support beyond the initial 2017-21 first term for these collaborations, for our Network to develop an online knowledge hub on respiratory diseases and a growing cohort of GHRN-fellows/GHRN-scholars. We encourage those interested in working with us, whether in the UK or internationally, to join our Network to improve the respiratory health of the world’s poorest populations.
